# Information Needs in Percutaneous Coronary Artery Intervention: Validation and Reliability Analysis of NPCI-10 Item Scale

**DOI:** 10.7759/cureus.12718

**Published:** 2021-01-15

**Authors:** Vasiliki Tsoulou, Georgios Vasilopoulos, Theodoros Kapadohos, George Panoutsopoulos, Antonia Kalogianni, Georgia Toulia, Ioannis Koutelekos, Georgia Gerogianni, Maria Polikandrioti

**Affiliations:** 1 Nursing, General Hospital Asklipieio Voulas, Athens, GRC; 2 Department of Nursing, University of West Attica, Athens, GRC; 3 Department of Nursing, University of Peloponnese, Sparta, GRC

**Keywords:** patient needs, heart failure, validation

## Abstract

Introduction: Percutaneous coronary intervention (PCI) is a nonsurgical procedure used in the treatment of coronary heart disease.

Purpose: The purpose of this study was to validate a scale created in order to assess the importance and fulfillment of information needs in patients after PCI.

Methods: A 10-item scale was created by the researchers to explore the level of information needs and the level of fulfillment of these needs. The total scores have a possible range of 10 to 40 with higher scores indicating higher importance and fulfillment. The validation of the questionnaire included face and content validity, construct validity, internal consistency, repeatability, and discriminant validity.

Results: Forty patients contributed to this validation. Mean and median scores for each question separately and also overall scores suggest that patients consider the need to be informed very important and that it was fulfilled to a very high degree (mean scores 39.5 and 39.3, respectively). All questions were found to be significantly correlated with the overall scores (rho > 0.3) meaning strong construct validity. Cronbach’s α coefficients were high (>0.7) indicating great consistency. Both total scores had great repeatability, which suggests a high degree of reliability of the participants' responses (ICCs > 0.8). Regarding discriminant validity, a statistically significant association was observed only between marital status and the degree of fulfillment of the need to be informed (p = 0.036). More specifically, divorced or widowed patients had a lower degree of fulfillment than married patients (mean 38.6 vs. 39.6).

Conclusion: It is a reliable instrument that will help clinicians who are at close contact with patients after PCI to gain a better understanding of their needs.

## Introduction

Cardiovascular disease remains the leading cause of mortality accounting for 31% of all deaths worldwide, and it is estimated to reach approximately 22.2 million deaths by 2030 [1,2]. More in detail, myocardial infarction morbidity and mortality rates are 1.5-15 times higher compared to general population [1].

However, surveillance of cardiac patients has increased due to improvements in pharmacological treatment and revascularization techniques. For example, percutaneous coronary intervention (PCI) has become one of the most widely performed procedures in cardiovascular medicine [3,4].

Therefore, needs of cardiac patients have come to the forefront of contemporary clinical practice. Impressively, provision of information to cardiac patients has slowly been acknowledged as a key component of therapy that promotes healthcare decision-making. It was several decades ago, in the late 1960's, when a shift from "paternalism" to patients' participation in therapeutic regimen was observed. Thereafter, information was gradually been applied in systematic clinical practice across the world.

Interestingly, information is associated with several benefits such as adherence to medical recommendations and modification of health-related behavior, alleviation of anxiety and depression, and commitment in attendance of scheduled follow-up in cardiology departments. Cardiac patients need accurate information about their health state, symptom management, or practical daily issues, thus avoiding suspicion and doubt or insecurity about treatment. Likewise, lack of information is triggering dissatisfaction, complaints, and several medical malpractices [5-8].

Therefore, the most crucial point is to understand cardiac patients' perspectives about their needs. Information represents what the patient wants to know from health professionals in order to cope effectively with the disease. This approach is associated with satisfaction of provided care and better long-term clinical outcomes.

Nowadays, patients have become more active, decisive, and assertive consumers of health care, which in turn creates the demand of evaluating and fulfilling their information needs in all phases of a disease. From patients' perspectives, assessment of information may demonstrate gaps that need to be incorporated in education. Meanwhile, clinicians have to confront with barriers that undermine the provision of effective information, such as non-availability of personnel, time, or suitable and validated instrument [5-10].

Therefore, the aim of this study was to validate the reliability of a short scale evaluating information needs of patients who underwent PCI (NPCI-10 item scale).

## Materials and methods

The sample of the study consisted of 40 outpatients who underwent PCI with drug-eluding stents. In the present study the convenience sampling method was used. Patients visited outpatient clinics for routine scheduled follow-up. This clinic was located in a tertiary university hospital in Athens. The study lasted from January 2020 to October 2020.

Criteria for patients’ inclusion in the study were as follows: (i) age above 18 years; (ii) PCI with drug-eluding stents; (iii) ability to write, read, and understand the Greek language; and (iv) the ability to read and sign the informed consent form. The exclusion criteria were as follows: (i) patients with a history of mental illness; (ii) patients visiting clinics to treat some other co-existing disease; and (iii) patients with cognitive disorders and sight or hearing problems.

Procedure

PCI patients that agreed to participate in the study were invited to a private room, which enabled participation to be performed in privacy and safety. The process of filling out the questionnaires lasted for 15 min and took place after patients had completed their follow-up in the outpatient clinic.

Ethical considerations

Written informed consent for participation in this study was obtained from all patients after explanation of the purpose and procedure of the study. Participation in the study was on a voluntary basis, and anonymity was preserved. Furthermore, all participants were informed of their right to refuse or to discontinue their participation, according to the ethical standards of the Helsinki Declaration of 1983. The study was approved by the Medical Research Ethics Committee of the hospital.

Instrument 

A 10-item scale was created by the researchers in order to assess information needs in patients who underwent PCI. These statements were selected taking into account the Questionnaire "Needs of hospitalized patients with coronary artery disease" 10 as well as the literature review. More in detail, patients were asked to answer two questions in each item of 10 in the scale of information needs: (a) how important did they perceive each of the items and (b) to what extent each item of information was fulfilled in clinical practice.

A four-point Likert scale was used to answer all items in the scale. The answers to the four-point scale were: Not at all, A little, Very, and Very much. The answers to the 10-item scale lead to two final scores: (a) the level of importance of the information need and (b) the level of fulfillment of the information need, using the sum separately for the answers concerning the importance and separately for the answers concerning the fulfillment. The total scores have a possible range of 10-40. Higher scores indicate higher level of importance and a higher level of fulfillment. The NPCI-10 item scale is self-administered and does not include reverse-scored items.

Since this scale was created by the researchers, the necessary reliability and validity of the tool were assessed. Initially, the questionnaire immediately after its construction was completed by five patients in order to determine whether the questions are comprehensible and clear (face validity). In addition, the same patients and five health professionals who had long experience in percutaneous coronary intervention (PCI) were asked to judge the content of the scale (content validity). All stated that the items were highly representative of the information needs (importance and fulfillment).

Validation of the NPCI-10 item scale

The construct validity of the scale was assessed by comparing the score of each statement with the total score of the scale separately for the importance of the information need and for the level of fulfillment. The comparison was performed using Spearman’s rho correlation coefficient. The coefficient takes values between -1 and +1. Values close to +1 indicate high structural validity.

The internal consistency (reliability) of the scale was assessed by calculating the Cronbach’s alpha index. This index ranges from 0 to 1. Large values of the alpha index indicate a large coherence of the questions that make up the subscale, hence high reliability. The Cronbach’s alpha, if item is deleted, was used to identify questions that reduced the internal consistency of the scale and therefore should be excluded.

The test-retest was performed applying the statistical criterion of intra-class correlation coefficient (ICC). To perform the repeatability test, the participating patients completed the scale for the second time 14 days after the first completion. This criterion takes values between -1 and +1. Values close to +1 indicate high repeatability of the scale. The results for the repeatability test are presented with the ICC index and 95% confidence intervals (CI).

The discriminant validity of the scale was assessed using basic statistical criteria to compare the final scores between the patients' characteristics. The statistical tests were the independent sample t-test and the analysis of variance (ANOVA) criterion.

Finally, the scores of each statement and the total scores are presented with means and standard deviation as well as with medians and interquartile range. The patient characteristics are presented with absolute and relative frequencies (%).

The observed statistically significant level was set to a = 5%. Analysis was performed with SPSS v25 (SPSS Inc., Chicago, USA).

## Results

Description of the sample

Table 1 presents the description of the sample. The majority of patients were men (85.0%), above 70 years (35.0%) and married (67.5%). Furthermore, 55% of the participants were pensioners, 45% had high-school education, and 50% had two children. Regarding the place of residence, it was found that 65% came from the wider region of Attica.

**Table 1:** Description of the sample (n = 40)

	n (%)
Gender	
Male	34 (85.0%)
Female	6 (15.0%)
Age	
41-50	7 (17.5%)
51-60	7 (17.5%)
61-70	12 (30.0%)
71-80	14 (35.0%)
Marital status	
Married	27 (67.5%)
Single	1 (2.5%)
Divorced/Widowed	12 (30.0%)
Education	
Primary school	15 (37.5%)
High school	18 (45.0%)
University	7 (17.5%)
Job	
Employee	15 (37.5%)
Unemployed/Household	3 (7.5%)
Retired	22 (55.0%)
Residence	
Attica	26 (65.0%)
County capital	9 (22.5%)
Small town/Village	5 (12.5%)
Children	
0	4 (10.0%)
1	10 (25.0%)
2	20 (50.0%)
> 2	6 (15.0%)

Description of scores

Table 2 describes the scores for the individual questions as well as the overall scores separately for the degree of importance of the need to be informed as well as the degree of fulfillment. We observe that since the mean and median scores are very close to the maximum of the possible range of answers for each question separately and also for the overall scores, this suggests that patients considered the need to be informed very important and that it was fulfilled to a very high level.

**Table 2:** Description of the scores (N = 40) SD: Standard Deviation, IQR: Interquartile range

	Importance		Fulfillment	
Statements (Range 1-4)	Mean (SD)	Median (IQR)	Mean (SD)	Median (IQR)
1. I would like to be informed of how much responsible I am for my current PCI state of health	3.93 (0.27)	4 (4-4)	3.88 (0.33)	4 (4-4)
2. I would like to be informed of how I can self manage my PCI in order to improve my health	3.98 (0.16)	4 (4-4)	4.00 (0.00)	4 (4-4)
3. I would like to be informed about my exact follow-up	4.00 (0.00)	4 (4-4)	3.98 (0.16)	4 (4-4)
4. I would like to be informed of every treatment I receive	3.98 (0.16)	4 (4-4)	3.98 (0.16)	4 (4-4)
5. I would like to be informed about prognosis of my PCI	3.93 (0.27)	4 (4-4)	3.90 (0.30)	4 (4-4)
6. I would like to be informed about the impact of PCI to my professional life	3.98 (0.16)	4 (4-4)	3.90 (0.30)	4 (4-4)
7. I would like to be informed about the impact of PCI to my social life	3.93 (0.27)	4 (4-4)	3.93 (0.27)	4 (4-4)
8. I would like to be informed about the necessary lifestyle changes due to PCI	3.93 (0.27)	4 (4-4)	3.93 (0.27)	4 (4-4)
9. I would like to receive written information about my PCI health (reason for re-admission, examinations, medications)	3.95 (0.22)	4 (4-4)	3.88 (0.33)	4 (4-4)
10. I would like to know that I can contact clinicians to be informed about my PCI	3.95 (0.22)	4 (4-4)	3.93 (0.27)	4 (4-4)
Total Score (Range 10-40)	39.53 (1.24)	40 (39.5-40)	39.28 (1.45)	40 (39-40)

Construct validity

Table 3 presents the results of the construct validity. All sub-questions were found to be statistically significantly correlated with the overall scores (p values < 0.05) with correlation coefficients rho > 0.3 indicating moderate to strong correlation. Meaning that, all questions provide important information in calculating the final score.

**Table 3:** Construct validity

	Total Score of Importance		Total Score of Fulfillment	
	rho	p value	rho	p value
Statement 1	0.549	0.001	0.592	0.001
Statement 2	0.339	0.032	-	-
Statement 3	-	-	0.314	0.049
Statement 4	0.357	0.024	0.314	0.049
Statement 5	0.549	0.001	0.502	0.001
Statement 6	0.357	0.024	0.473	0.002
Statement 7	0.559	0.001	0.529	0.001
Statement 8	0.354	0.025	0.490	0.001
Statement 9	0.505	0.001	0.424	0.006
Statement 10	0.499	0.001	0.424	0.006

Reliability-Internal Consistency

Table 4 presents the results of the internal consistency. We conclude that the internal consistency of the questions that take part in the overall score (degree of importance and degree of fulfillment) is extremely high (Cronbach’s α > 0.7), which indicates great consistency and reliability of the participants' answers.

**Table 4:** Internal consistency

	Cronbach’s α
Total Score of Importance	0.781
Total Score of Fulfillment	0.756

Discriminant validity

Table 5 presents the scores of importance and fulfillment with respect to characteristics of patients. A statistically significant association was observed only between marital status and the degree of fulfillment of the need to be informed (p = 0.036). More specifically, divorced or widowed patients had a lower level of fulfillment than married patients (mean 38.6 vs. 39.6). The other characteristics were not found to be significantly associated with the scores.

**Table 5:** Discriminant validity

	Total Score of Importance		Total Score of Fulfillment	
	Mean (SD)	p value	Mean (SD)	p value
Gender		0.687		0.158
Male	39.56 (1.31)		39.41 (1.48)	
Female	39.33 (0.82)		38.50 (1.05)	
Age		0.638		0.529
≤60 years	39.71 (0.83)		39.29 (1.14)	
61-70 years	39.25 (2.01)		38.92(2.23)	
>70 years	39.57 (0.65)		39.57 (0.76)	
Marital status		0.089		0.036
Married	39.73 (0.67)		39.62 (0.75)	
Divorced/Widowed	39.00 (2.00)		38.58 (2.27)	
Education		0.418		0.197
Primary school	39.60 (0.63)		39.53 (0.64)	
High school	39.28 (1.74)		38.83 (2.01)	
University	40.00 (0.00)		39.86 (0.38)	
Job		0.397		0.919
Employee	39.73 (0.80)		39.27 (1.10)	
Retired	39.36 (1.53)		39.32 (1.73)	
Residence		0.954		0.893
Attica	39.46 (1.48)		39.15 (1.69)	
County capital	39.56 (0.73)		39.44 (0.88)	
Small town/Village	39.75 (0.50)		39.50 (1.00)	
Children		0.155		0.144
0	39.75 (0.50)		38.75 (1.50)	
1	39.90 (0.32)		39.70 (0.48)	
2	39.60 (0.82)		39.50 (0.89)	
>2	38.50 (2.74)		38.17 (3.06)	

## Discussion

Scales are the most widely used tools due to the low cost and their ease in applying, and they provide a context to report all informational topics.

Evaluation of the level of importance and the level of fulfillment of information needs is limited, though various studies highlight the importance of measuring the patients’ learning needs. To the best of our knowledge the most widespread instrument among cardiac patients is the "Cardiac Patients Leaning Needs Inventory (CPLNI)," which was developed by Gerard and Peterson in 1984. The CPLNI contains 43 items, organized in eight domains (Introduction to the Critical Care Unit, Anatomy and Physiology, Psychological Factors, Risk Factors, Medication Information, Diet Information, Physical Activity, and Other Pertinent Information). However, in the present we did not use CPLNI as a gold standard since we focus on different dimensions especially focused on PCI intervention [11,12].

The present 10-item scale is a tool for better understanding of PCI patients' needs, which is at the same time applied within short time. This scale can be used both in interview setting and as a self-report instrument. It is reliable and presents satisfactory validity, making it suitable for use in research and clinical settings. Additionally, PCI per se demands evaluation of patients' needs. For example, receiving prescribed antiplatelet and other secondary preventive medication after PCI needs constant evaluation. It is extremely important to follow this medication regimen for at least one year to avoid possible complications related to stent implantation.

Moving toward a more patient-centered care aims to maximize patients’ self-care abilities to understand of illness-related events and to ensure that procedure will be successful, thus minimizing healthcare expenditure rises and improving their quality of life [13-15].

Nowadays, as short hospital stay after PCI (many patients are able to go home the day following the procedure) has limited the opportunities for clinicians to provide pre-discharge information, the challenge of assessing and meeting patients’ information needs becomes apparent [16].

Needs of patients after PCI merit deep exploration as Perk et al. [17] declared in their study. More in detail, researchers showed that 67% of participants (n = 1.073) perceived they were cured, 38% perceived there was no need to change their habits, 16% continued to use tobacco, and only 27% reported that they still had cardiovascular disease and needed behavioral change. Though nutritional counseling was provided to 71%, only 40% managed to change food habits. Evaluation of information needs can potentially identify patients at high risk of adverse clinical outcomes, thus minimizing healthcare utilization and increased cost.

Patients' perceptions of their educational needs differ from the perceptions of physicians, nurses, and relatives of the patients [18,19]. Kilonzo et al. [18] who explored 33 PCI patients and 13 nurses showed that disease-specific items, physical action, psychosocial, and emotional information were the categories that patients found most important. Cardiac nurses focused more on psychosocial and emotional issues.

Gentz et al. [20] supported that learning needs in the acute care setting differ from those in the outpatient setting. Continuing informational resources need to be available for patients who are recovering from PCI.

Limitations of the study

This study has some limitations. We, however, acknowledge methodological caveats that should be considered when interpreting the results from this study. Convenience sampling is one of the limitations in this study. This method is not representative of all population living in Greece, thus limiting the generalizability of results. Additionally, there was no instrument use as a gold standard.

## Conclusions

Enhancing awareness about the importance of information needs after PCI would benefit thousands of cardiac patients around the world. In Greek clinical settings, there was a clear need for the development of a needs’ assessment tool, which is also valid and reliable for use among this growing population.

The instrument will help clinicians who are at close contact with patients after PCI to gain a better understanding of their needs and in turn to develop appropriate interventions to address such needs. Identifying information needs after PCI is essential to implement effective health education programs based on patient-centered care.
